# Recombination Hotspots Flank the *Cryptococcus* Mating-Type Locus: Implications for the Evolution of a Fungal Sex Chromosome

**DOI:** 10.1371/journal.pgen.0020184

**Published:** 2006-11-03

**Authors:** Yen-Ping Hsueh, Alexander Idnurm, Joseph Heitman

**Affiliations:** Department of Molecular Genetics and Microbiology, Duke University Medical Center, Durham, North Carolina, United States of America; Washington University, United States of America

## Abstract

Recombination increases dramatically during meiosis to promote genetic exchange and generate recombinant progeny. Interestingly, meiotic recombination is unevenly distributed throughout genomes, and, as a consequence, genetic and physical map distances do not have a simple linear relationship. Recombination hotspots and coldspots have been described in many organisms and often reflect global features of chromosome structure. In particular, recombination frequencies are often distorted within or outside sex-determining regions of the genome. Here, we report that recombination is elevated adjacent to the mating-type locus *(MAT)* in the pathogenic basidiomycete *Cryptococcus neoformans.* Among fungi, C. neoformans has an unusually large *MAT* locus, and recombination is suppressed between the two >100-kilobase mating-type specific alleles. When genetic markers were introduced at defined physical distances from *MAT,* we found the meiotic recombination frequency to be ~20% between *MAT* and a flanking marker at 5, 10, 50, or 100 kilobases from the right border. As a result, the physical/genetic map ratio in the regions adjacent to *MAT* is distorted ~10- to 50-fold compared to the genome-wide average. Moreover, recombination frequently occurred on both sides of *MAT* and negative interference between crossovers was observed. *MAT* heterozygosity was not required for enhanced recombination, implying that this process is not due to a physical distortion from the two non-paired alleles and could also occur during same-sex mating. Sequence analysis revealed a correlation between high G + C content and these hotspot regions. We hypothesize that the presence of recombinational activators may have driven several key events during the assembly and reshaping of the *MAT* locus and may have played similar roles in the origins of both metabolic and biosynthetic gene clusters. Our findings suggest that during meiosis the *MAT* locus may be exchanged onto different genetic backgrounds and therefore have broad evolutionary implications with respect to mating-type switching in both model and pathogenic yeasts.

## Synopsis

It is hypothesized that sexual reproduction enables genetic recombination between individuals to generate diversity, thus increasing population fitness. However, a well-known meiotic feature is that recombination is not randomly distributed across the genome: “coldspots” and “hotspots” exist, implying some regions undergo exchange more frequently. In this paper, the authors report the discovery of recombination hotspots linked to the sex-determining region mating-type locus *(MAT)* in the human pathogenic fungus *Cryptococcus neoformans.* Through genetic analysis, hotspots were found to reside on both sides of *MAT* and are associated with DNA regions marked by high G + C base pair composition. Moreover, recombination on one side of *MAT* is associated with a recombination on the other. As a result, the *MAT* locus can be replaced onto the homologous chromosome as a unit—an effective switch of *MAT*. Based on these findings, they propose that the *MAT*-linked recombination hotspots impacted key steps during *MAT* evolution. This study has broader implications on how gene clusters (including those involved in metabolism or secondary metabolite production) are assembled and maintained and explains how recombination is distorted in sex-determining regions in eukaryotes.

## Introduction

Enhanced genetic recombination is a hallmark of meiosis and serves two basic functions. First, genetic reassortment is enhanced through crossing-over between the homologous maternal and paternal chromosomes, mixing the genetic constitution in gametes. Second, in most organisms crossing-over is critical for faithful chromosome segregation during meiosis by ensuring the generation of tension between sister chromosomes attached to opposite spindles.

Meiotic recombination events are not random and occur more frequently in some regions of the genome (recombination hotspots), where double-stranded DNA breaks (DSBs) are often preferentially induced. This is a universal phenomenon in many eukaryotes in which meiotic recombination has been studied [1]. Hotspots have been well studied in the budding yeast *Saccharomyces cerevisiae,* the fission yeast *Schizosaccharomyces pombe,* mouse, and human. Many of the molecular features associated with hotspots are conserved. For example, in each case DSB formation is initiated by the type II topoisomerase-related protein Spo11 without any particular sequence preference. Hotspots are usually small (between 1 to 3 kilobase [kb]), and there is often an association between chromatin structure and hotspot activity [2–4]. In *S. cerevisiae,* meiotic recombination hotspots and coldspots have been globally mapped using a microarray-based approach, and although there is no single characteristic shared by all hotspots, a high G + C base composition is significantly correlated with an increased occurrence of DSBs [3]. Moreover, studies from S. cerevisiae and S. pombe suggest that hotspots and coldspots might in fact reflect the presence of chromosomal features, such as chromatin structures that can affect the accessibility of DNA to the recombination machinery [4–6].

In fungi, recombination is often increased or decreased near or within a specialized region of the genome: the mating-type locus. Mating-type loci *(MAT)* are the sex-determining regions of fungal genomes [7] and serve as paradigms to study gene regulation and sexual development [8,9]. In a bipolar mating system, **a** and α mating types are controlled by a single *MAT* with two alternative alleles, *MAT* **a** and *MAT*α. To serve efficiently as a sex-determinant, *MAT* must be inherited as a single unit to prevent the generation of sterile or self-fertile offspring. As a consequence, recombination is suppressed within this region and mechanisms that contribute include extensive sequence divergence and rearrangement between different *MAT* alleles [10]. There are extreme examples, such as *Neurospora tetrasperma,* in which recombination is suppressed over a larger portion of the chromosome on which *MAT* resides (60%), but there is an obligatory crossover distal to the recombinationally suppressed region that ensures proper chromosomal segregation [11].

Another *MAT*-associated recombination feature is mating-type switching, which occurs in the hemiascomycetous *Saccharomyces sensu stricto* group and related species, including *Candida glabrata* and *Kluyveromyces lactis* [12]. In these species, haploid mother cells have the ability to undergo a mating-type switch, which generates cells of opposite mating type in close proximity to facilitate mating and return to the diploid state [13,14]. In *S. cerevisiae, MAT* and the silent cassettes *HML* and *HMR* are encoded on Chromosome III, and the silent cassettes are in subtelomeric regions and subject to transcriptional silencing by the Sir protein complex. Mating-type switching is the result of a nonreciprocal homologous recombination event (gene conversion) from *HML* or *HMR* to *MAT* induced by an Ho endonuclease-generated DSB at the active *MAT* cassette. More distantly related species such as K. lactis harbor silent mating-type cassettes but no Ho endonuclease, and mating-type switching occurs at lower efficiency via mitotic recombination [15]. This provides evidence that the silent *MAT* cassettes were acquired prior to the *HO* gene during evolution [12].

Studies in S. cerevisiae revealed that the sequence at the active *MAT* locus is replaced by copying new sequences from templates that reside elsewhere in the genome: namely at the silent mating-type cassettes *HML*α and *HMR* **a** [16,17]. Two aspects of this process are highly directional. First, *HML*α or *HMR* **a** serve as the donor of genetic information and *MAT* as the recipient. Second, ~90% of the time cells switch to an opposite mating type, avoiding non-productive switching from the other silent cassette. This is achieved via a 700-base pair (bp) recombinational enhancer (RE) that defines donor site preference [16,18]. In **a** cells, RE is occupied by the binding of the transcription factors Mcm1 and Fkh1 and activates the left arm of Chromosome III and *HML*α for recombination proficiency. In α cells, RE is inactivated by the mating-type regulator α2 encoded by *MAT*α, which directs *HMR* **a** to become the default donor [19,20].

In the more distantly related fission yeast *S. pombe,* a mating-type switching system operates, but its distinct organization and functional mechanisms reveal it evolved independently from the switching system of *S. cerevisiae.* In this example, two silent and one active *MAT* cassettes are closely linked in an ~30-kb region of the genome. Mating-type switching involves an unusual replication-coupled recombination event triggered by an imprint on the active *MAT* locus *mat1,* which may involve either a nick or ribonucleotides that remain from an RNA primer of an Okazaki fragment [21–24]. In the basidiomycete phylum of fungi, Agrocybe aegerita has been reported to switch mating type, although the mechanism is unknown [25]. These examples show that mating-type switching evolved independently in several different phylogenetic lineages of fungi.

Cryptococcus neoformans is a haploid basidiomycete human pathogen that serves as a model for both fungal pathogenesis and basic biology [26–28]. The whole genome sequences of two divergent varieties, var. *grubii* (serotype A) and var. *neoformans* (serotype D), which diverged ~20 million years ago and have different virulence properties, are publicly available and have significantly advanced our understanding of this pathogen [29]. The sexual cycle of this organism is well defined and involves a bipolar mating system with **a** and α cells [30,31]. Because the spores produced during mating may represent the infectious propagule of *C. neoformans,* research on the sexual cycle has been one of the main focuses [32,33]. C. neoformans lacks silent mating-type cassettes and does not switch mating type. Moreover, the *MAT* locus of C. neoformans is unusually large, spanning more than 100 kb and containing more than 20 genes, including those involved in establishing cell type identity (homeodomain proteins), pheromone production and sensing, and others whose functions during mating, if any, are unknown [10,34]. Extensive sequence rearrangements between the **a** and α alleles are hypothesized to inhibit recombination within *MAT* to prevent formation of hybrid alleles or dicentric and acentric chromosomes.

Here we report that recombination hotspots flank the borders of *MAT* in both serotype A and D strains of C. neoformans. These hotspots are associated with regions with a high G + C base composition. We discuss hypotheses concerning their possible roles in the origin and evolution of the *MAT* gene cluster in this important human fungal pathogen and discuss the relationship of these discoveries to our understanding of the mating-type switching paradigms in budding and fission yeast. We consider a recently discovered process (D. Soll, personal communication), by which the *MTL* in *Candida albicans* undergoes homozygosis via a localized gene conversion event (presumably as a prelude to mating), illustrating a second example of mating-type-associated recombination in another common human fungal pathogen. Finally, we discuss the implication of our findings for the evolution of other gene clusters involved in metabolism or the biosynthesis of nature products.

## Results

### The Recombination Frequency around the *MAT* Locus Is Unusually High

An RFLP-based genetic linkage map of C. neoformans was recently reported for two serotype D var. *neoformans* strains whose entire genomes have been sequenced [29,35]. We noticed that the genetic map was expanded adjacent to the *MAT* locus, suggesting the existence of recombinational hotspots. To characterize the recombination frequency around the *MAT* locus, serotype A strains bearing dominant drug resistance markers at defined distances from the *MAT* locus were constructed and crossed to wild-type strains of opposite mating type. Basidiospore meiotic progeny were randomly isolated with a micromanipulator, germinated, and the resulting yeast colonies were scored for mating type by crossing to tester strains and for the dominant markers conferring drug resistance on selective medium.

**Figure 1:**
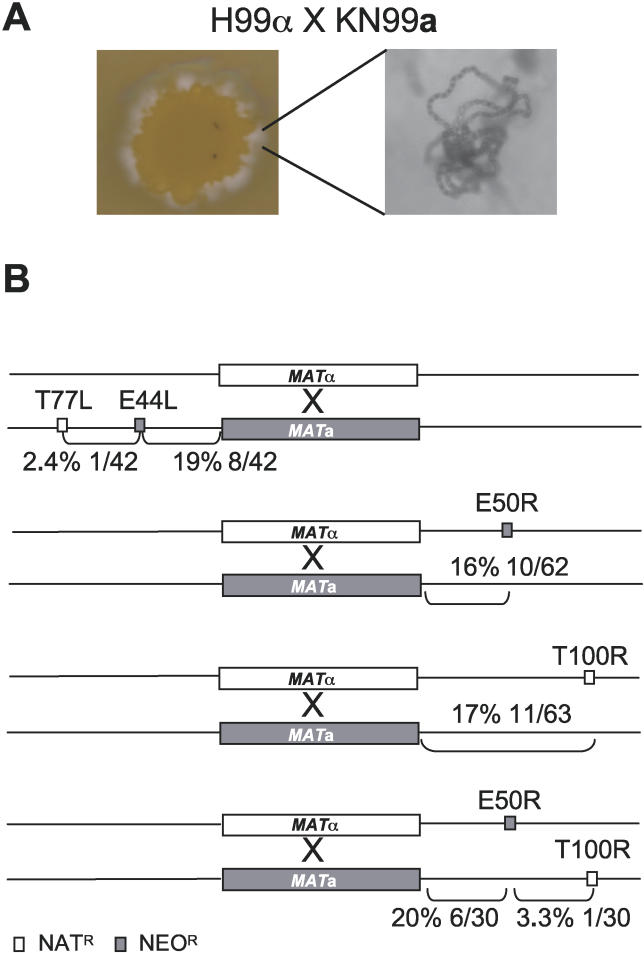
The Recombination Frequency around the *MAT* Locus Is Unusually High (A) The mating of C. neoformans is shown. Left, cells of opposite mating type were mixed on V8 mating medium, and the white filaments around the yeast colony edge are dikaryons produced by mating. Right, chains of basidiospores observed by microscopy. (B) The meiotic recombination frequency between *MAT* and flanking markers is indicated in % and as the number of recombinants in the total progeny analyzed. The first letter of the marker indicates the dominant selectable marker (E for NEO^R^ and T for NAT^R^); the following number indicates the approximate distance between the marker and the *MAT* boundary; and the last letter indicates the relative position of the marker to the *MAT* locus (L for left and R for right).

This segregation analysis revealed an elevated level of recombination adjacent to *MAT.* The recombination frequency between the *MAT* locus and an inserted NEO^R^ marker (E), which lies 44 kb from the telomere proximal (left) end (E44L) or 50 kb from the telomere distal (right) end (E50R), was 26% and 16% (Figure 1; unpublished data). In other words, in this region of the genome the relationship between the genetic and physical maps is ~2.2 kb/cM (~0.45 cM/kb), which is ~6 times greater recombination than the genome average (13.2 kb/cM or ~0.076 cM/kb) [35].

To elucidate whether the elevated recombination frequency observed is truly *MAT-*associated, strains were constructed in which two dominant markers were inserted at different distances in the left flanking region of *MAT* (NAT^R^-NEO^R^-*MAT*). This allowed us to measure recombination between NAT^R^ and NEO^R^, between NEO^R^ and *MAT,* and between NAT^R^ and *MAT.* A NAT^R^ marker (T) was inserted 77 kb from the left boundary (T77L) of the strain harboring the E44L marker and the recombination frequency between *MAT* and the NEO^R^, and the NAT^R^ marker was then scored. In contrast to the high recombination frequency between *MAT* and the NEO^R^ marker, which is proximal to *MAT* compared to the NAT^R^ marker, the crossover rate between the two dominant markers that are separated by a similar physical distance between *MAT* and NEO^R^ was significantly lower (2.4%, Figure 1). In addition, this analysis also suggests that the recombination events detected are more likely to be crossover events instead of gene conversion at the E44L marker because ~97% of the progeny were either NEO^R^ and NAT^R^ or NEO^s^ and NAT^s^, indicating a single recombination event between *MAT* and the proximal dominant marker.

A *MAT* **a** strain carrying a NAT^R^ marker inserted 100 kb from the right boundary (T100R) was generated and crossed to either a *MAT*α wild-type or *MAT*α E50R strain to score the recombination frequency. Similar to the observations on the *MAT* left boundary, the recombination frequency between *MAT* and either NEO^R^ or NAT^R^ integrated in the right flanking region was high (16% and 17%) in both crosses, and was significantly higher than the frequency between the two dominant markers (3.3%). In the 50-kb region between the two dominant markers, the ratio between the genetic and physical maps is close to the whole genome average (15.0 kb/cM or 0.067 cM/kb).

Thus, based on these segregation analyses in the *MAT* flanking regions, we conclude that the recombination frequency on both sides of *MAT* is significantly higher than the genome average, implying the presence of a mechanism that enhances recombination in this region of the genome.

### Crossover Events Occurred Close to the *MAT* Borders during the Isolation of Congenic Strains

Previous studies have provided evidence that crossing over is suppressed across the *MAT* locus during meiosis [10], but the discovery of regions with a high level of recombination near *MAT* suggested that crossover events may readily occur in the regions just outside of the *MAT* locus. To detect these events in a natural setting, and without the introduction of foreign DNA that could influence recombination, we examined when and where crossovers occurred during the generation of the congenic serotype A var. *grubii* strains H99 and KN99**a** [36].

**Figure 2:**
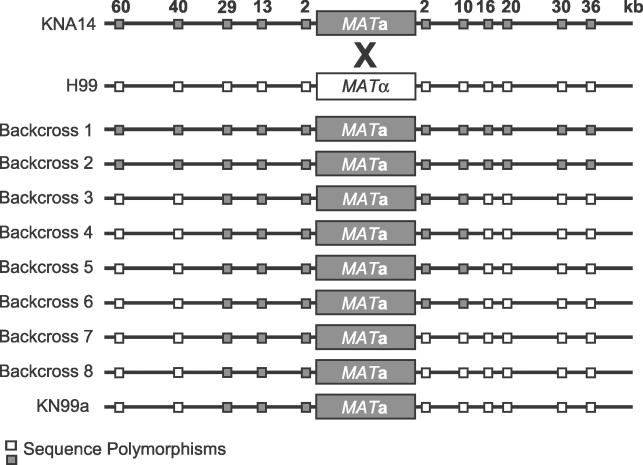
Detection of Crossover Events That Occurred during the Isolation of Congenic Strains The gray boxes represent the *MAT* **a** locus and sequences specific to strain KNA14 and the white boxes represent the *MAT*α locus and sequences specific to strain H99. The numbers indicate approximate distances between the polymorphisms and the left or right boundaries of the *MAT* locus. These polymorphisms were monitored in each backcross generation and the final congenic strain KN99**a**.

More than 20 different polymorphisms between the parental strains H99 and KNA14, encompassing the 60-kb regions on both sides of the *MAT* locus, were identified with different approaches (see Materials and Methods). These regions were then amplified by PCR and sequenced in each of the backcross strains. During the generation of these congenic strains, one crossover occurred on the telomere-proximal (left) side and two crossovers occurred on the telomere-distal (right) side of *MAT* (Figure 2). Double crossovers, one on each side of the *MAT* locus, were observed in backcross 3. One of the crossovers lies between 40 kb and 29 kb from the left border of *MAT,* and the other lies between 10 kb and 16 kb from the right border. We assume that these were crossover recombination events, because polymorphic markers beyond the recombining region, which span more than 20 kb, all have the same allele. A second crossover event occurred within the 2-kb region from the right border of the *MAT* locus in backcross 6, and as a consequence, the remaining polymorphisms 3′ to this junction became homozygous.

In addition to the H99 and KN99**a** congenic strain pair, sequence polymorphisms in the var. *neoformans* congenic strain JEC20 and JEC21 were also analyzed. Similar to those described above, the polymorphisms were distributed in a ~30-kb interval in the left flanking region and in a ~6-kb interval in the right flanking region of *MAT.* This result also suggests crossover events occurred closer to the *MAT* boundary at the telomere-distal end of *MAT.* We also examined the *MAT* adjacent region in two more recently isolated congenic var. *neoformans* strain pairs KN3501**a**/KN3501α and KN433**a**/KN433α [37]. Interestingly, in contrast to the JEC20 and JEC21 strain pair, in both cases crossover events occurred closer to *MAT* on both flanks based on the RFLP markers used in that study [37].

The result of crossover mapping in the backcross strains shows that in the absence of introduced exogenous DNA, recombination has occurred at a high frequency in regions adjacent to both sides of *MAT* in both serotype A and D strains. Furthermore, the potential hotspots in H99 might be located between 29 kb to 40 kb from the left *MAT* border and between either 10 kb to 16 kb or within 2 kb from the right *MAT* border.

### Deletion of the Potential Activator Sequence at the Right Border of the *MAT* Locus

**Figure 3:**
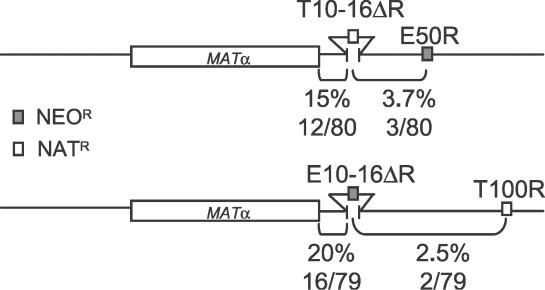
Deletion of the Potential Hotspot at the Right Border of the *MAT* Locus Revealed the Hotspot Lies Closer to the *MAT* Boundary The DNA in the 10- to 16-kb region from the right boundary of *MAT* was replaced with the NEO^R^ or NAT^R^ dominant markers and the resulting genetically marked strains were crossed to wild-type. Recombination frequency in the progeny analyzed is indicated as a percentage.

To test the hypothesis that a recombinational activator might reside in the 10- to 16-kb interval on the right border of the *MAT* locus, this 6-kb interval was deleted and the recombination frequency was assayed. In strains E50R and T100R, the 6-kb region was deleted and replaced with the NAT or NEO resistance marker (T10–16ΔR and E10–16ΔR) through biolistic transformation and homologous recombination (Figure 3). The resulting strains were crossed to wild-type cells of opposite mating type and the recombination frequency was scored by segregation analysis, as described above. If the recombinational activator were located in this region, the recombination frequency between the *MAT* locus and the flanking distal dominant markers should decrease significantly. However, we still observed close to 20% recombination between *MAT* and the dominant marker that is located ~10 kb away from the right border, indicating that the recombination hotspot lies even closer to *MAT* than initially hypothesized.

### Fine Mapping Suggests a Recombinational Activator Is Located in the 1- to 5-kb Interval from the Right *MAT* Border

**Figure 4:**
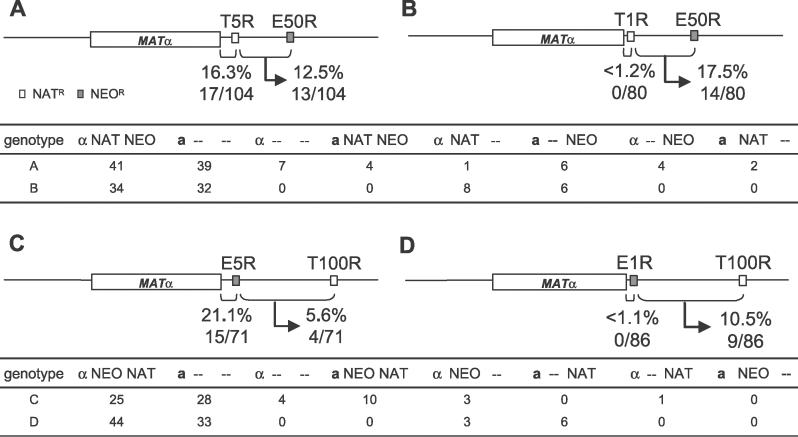
Fine Mapping Defines a Hotspot Located in the 1- to 5-kb Interval from the Right *MAT* Border Strains harboring two dominant selectable markers at the indicated positions were generated and crossed to the wild-type strain KN99**a**. Recombination frequencies between *MAT* and the proximal markers and between the proximal and distal markers were scored and depicted as percentages. The number of progeny in each genotype category is indicated in the table. More recombination events were detected between *MAT* and the distal marker when the proximal marker is located 5 kb from *MAT* rather than 1 kb. We think that the marker insertion at 1 kb is likely to interfere with the activity of the hotspot.

The mapping results showed that the crossover rate is still high between *MAT* and dominant markers integrated 10 kb away (T10R or E10R). We therefore integrated dominant selectable markers even closer to the border of the *MAT* locus to narrow the window of the crossover hotspot. Strains carrying the NAT (T) or NEO (E) resistance marker at 1 kb or 5 kb from the right border of the *MAT* locus (T5R, T1R, E5R, and E1R) were generated in strains that already bore a different dominant marker inserted at 50 kb or 100 kb from the *MAT* border (Figure 4). Following their construction, these strains were crossed to wild-type mating partners and independently scored for their meiotic recombination frequencies.

Based on this analysis, the T1R and E1R markers are tightly linked to the *MAT* locus and no recombinant was recovered from 86 or 80 progeny. However, the T5R and E5R markers still exhibited 15% to 20% recombination with *MAT* (Figure 4) similar to the level observed when the markers were inserted at the 10-kb and 50-kb positions. This result indicates that the physical/genetic map ratio in this 5-kb region adjacent to *MAT* is 50 times less than the genome-wide average (0.27 kb/cM versus 13.2 kb/cM or 3.7 cM/kb versus 0.076 cM/kb), implying that recombination occurs at a 50-fold higher rate per bp. This observation supports the conclusion that one of the *MAT*-associated hotspots is located between 1 kb to 5 kb from the right junction of the *MAT* locus. This conclusion is also in accord with previous mapping results in the backcross strains, in which a second crossover event was detected within the 2-kb interval from the right junction of the *MAT* locus.

We attempted to delete this 4-kb region to prove that it is required for the hotspot activity. However, no deletion mutants were obtained from 102 transformants screened. This may be due to the fact that the *NOG2* gene, which resides in this region of the genome, is an essential nucleolar GTPase in S. cerevisiae and may therefore also be essential in *C. neoformans.* Alternatively, the *NOG2* and neighboring gene *HOT1,* also within the 4-kb deletion, could together be essential.

### Crossover on One Side of *MAT* Is Associated with Crossover Events on the Other

Both genetic and cytological experiments have demonstrated the existence of an interference effect on meiotic recombination in many organisms as diverse as flies, plants, yeast, and humans [38–40]. The occurrence of one crossover often greatly reduces the probability of a second event in close proximity. On the other hand, a negative interference effect, which is less common, describes the situation in which an excess of double crossovers is observed in adjacent intervals relative to the crossover rate of each region. Based on our analysis of meiotic recombination in *C. neoformans,* two pieces of evidence suggested that hotspots might exist on both sides of the *MAT* locus. First, segregation analysis showed that markers placed just outside either side of *MAT* have a higher recombination frequency compared to the genome average (Figures 1, 3, and 4). Second, we detected crossover events that occurred during the construction of the serotype A congenic strain pair on both sides of *MAT* (Figure 2).

**Figure 5:**
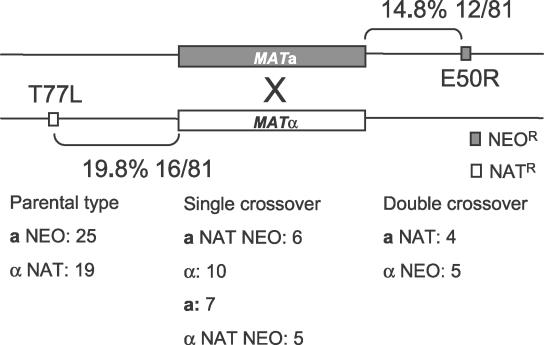
Crossing Over on One Side of the *MAT* Is Associated with Crossover Events on the Other Side Strains bearing markers on opposite sides of *MAT* were crossed and progeny were analyzed. The number of parental-type progeny, SCO progeny, and DCO progeny is indicated; DCO events occurred at a higher frequency than expected.

To investigate whether a crossover event on one side of *MAT* interferes with the probability of crossing over on the other side (an interference effect), strains bearing the T77L and E50R markers were crossed (T77L α × **a** E50R), and germinated basidiospores were analyzed for their mating type and NAT or NEO drug resistance. Of the 81 progeny examined, 16 experienced a single crossover (SCO) on the left side and 12 on the right side of *MAT*. In addition, nine progeny underwent a double crossover (DCO) on both sides of *MAT*. As shown in Figure 5, the SCO frequencies between T77L/*MAT* and *MAT*/E50R are 19.8% and 14.8%, respectively. Based on this result, if the occurrence of crossover events on both sides of *MAT* were to be independent, the expected frequency of a DCO occurring is ~2.9% (0.198 × 0.148). However, if interference affected the occurrence of a DCO event, then the frequency would be expected to be even lower than 2.9%. In marked contrast, in the set of 81 progeny we observed that 11.1% underwent a DCO during meiosis; four are **a** T77L and five are α E50R. This frequency is almost four times higher than expected, and therefore indicates that instead of a commonly observed interference effect, a negative interference is observed in the *MAT* flanking regions during meiotic recombination.

Most classic studies on interference effects have been conducted in *Drosophila* and instead of multiplying the SCO frequencies of both intervals, map units including all of the recombination events (both SCO and DCO) are multiplied to calculate the frequency of expected DCO events. In most of these cases, the impact of DCO events on map distance is very small (<1) because there are relatively few events; thus, inclusion of DCO events does not have a significant impact on the analysis. However, in our study, because the number of events in the DCO category is significantly larger (11), if we analyzed our data in this fashion, the expected DCO frequency would be 8% (19.8% + 11%) × (14.8% + 11%), which is closer to the level detected (11%). In other words, any negative interference effect would be less prominent if DCO events are included in the calculation.

To summarize, DCO events frequently occur in the flanking regions of the *MAT* locus during meiosis and serve to exchange the *MAT* locus onto a different genetic background.

### *MAT* Heterozygosity Is Not Required for Recombination Activation

C. neoformans has an unusually large *MAT* locus for a fungal species (~120 kb) and this specialized genomic region is characterized by nucleotide divergence and numerous inversions and transpositions that suppress recombination across the locus [10,34]. Presumably, this region of the genome forms an unpaired DNA segment during meiosis, similar to observations of the mating-type-like chromosome in N. tetrasperma [11].

To take these special features of the *MAT* locus into account, two alternative models were considered to explain the enhanced recombination frequency adjacent to *MAT*. In the first model, *cis-*activator sequences flank the *MAT* borders and enhance recombination. In the second model, the unusual genomic structure presented by *MAT* heterozygosity promotes crossing over on both sides of *MAT*. To test the latter hypothesis, homozygous diploid **a**/**a**, α/α, and **a**/α strains were constructed that bear a T50L marker on one of the *MAT*-chromosomes and an E50R allele on the other. Based on previous studies, meiotic sporulation is relatively less efficient in the **a**/**a** or α/α homozygous diploids compared to **a**/α heterozygous diploids, and an ectopically expressed homeodomain protein from the opposite mating type promotes sporulation of *MAT* homozygous strains [28]. Therefore, we expressed the Sxi1α homeodomain protein in the **a**/**a** diploid strain and Sxi2**a** in the α/α diploid strain, enabling the formation of the Sxi1α/Sxi2**a** heterodimer to promote meiosis and sporulation in the *MAT* homozygous diploid backgrounds [28,41].

**Figure 6:**
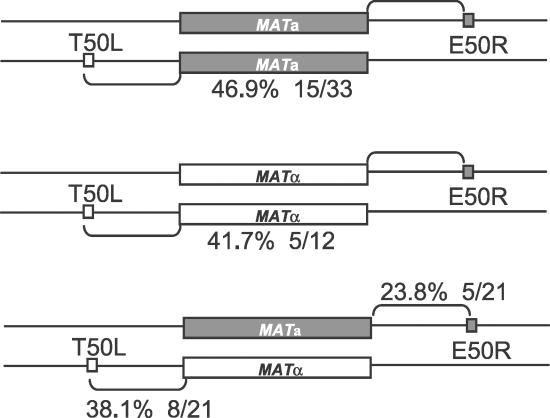
*MAT* Heterozyosity Is Not Required for Recombination Activation *MAT* homozygous and heterozygous diploid strains carrying one dominant marker on each side of *MAT* were generated and induced for meiotic sporulation by introduction of the *SXI1α* or *SXI2* ***a*** genes. Each haploid spore was isolated and the recombination frequency between *MAT* and the dominant markers was analyzed.

A high frequency of recombination was observed in both *MAT*-heterozygous and *MAT*-homozygous diploid strains (Figure 6). In the **a**/**a** diploid strain, ~45% of the progeny underwent a crossover event between *MAT* and one of the flanking dominant markers. These progeny now carry either both or neither of the drug resistance markers. If we assume that one-half of the crossovers occurred between *MAT* and T50L and the other one-half occurred between *MAT* and E50R, then the crossover rate is ~23% on each border of *MAT*. This recombination frequency is somewhat higher than that observed in an **a** × α cross and could be attributable to recombination within the two homozygous *MAT* alleles. Similar results were also observed in the α/α diploid strain where a recombination frequency of ~21% was observed on both sides of *MAT.* We noted that in both cases, the actual recombination frequency could be higher than that detected, because our experimental design is unable to distinguish between DCO events and parental-type progeny.

Based on these observations, we conclude that *MAT* heterozygosity is not required for *MAT*-associated recombination. In addition, the recombinational activators are unlikely to reside exclusively within either the α or the **a** allele of the *MAT* locus itself because we observed a similar level of recombination in both the **a**/**a** and the α/α diploid strains. We therefore favor the alternative model in which *cis-*activator sequences flank the *MAT* locus and enhance recombination. This conclusion also leads to the implication that these *cis-*activator sequences may also function during the same-sex mating process.

### Congruence of Hotspots and G + C Rich Regions Flanking *MAT*

**Figure 7:**
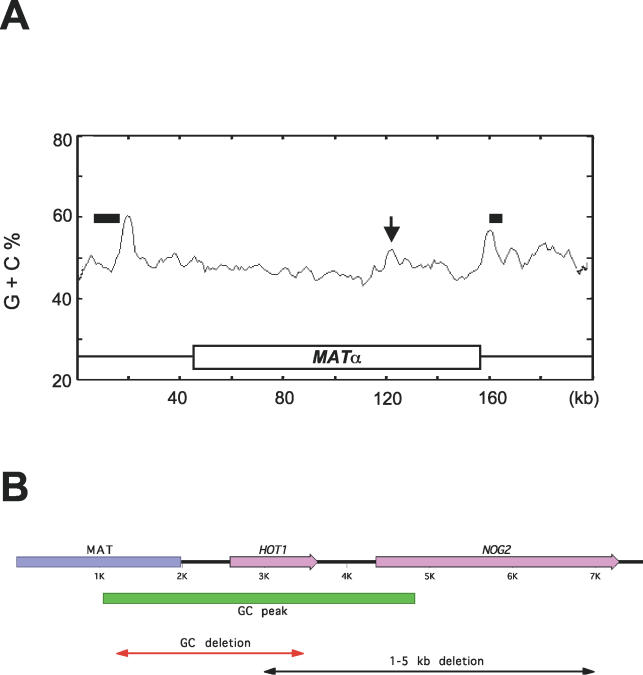
The Hotspots Are Associated with a High G + C Content (A) The sequence of the *MAT* locus along with 45 kb upstream and 40 kb downstream of *MAT* from strain H99 was plotted for G + C base composition with a 4-kb moving window. The thick lines represent the potential hotspot locations on both sides of *MAT* and the arrow indicates the minor G + C peak in the intergenic region between the divergently transcribed *RPO41* and *BSP2* genes. (B) The G + C peak is constituted of the terminal sequence of *MAT,* a predicted gene *HOT1,* and the N-terminal sequence of *NOG2.* The left arrow indicates the 60% of the G + C rich sequence that was deleted and replaced with marker T0R and the right arrow indicates the 4-kb region that was intended to be deleted and contains a possible essential gene *NOG2.*

Based on known mechanisms that promote recombination, hotspots can be classified into three different non-mutually exclusive categories [5]. α hotspots require transcription factor binding, β hotspots are associated with nucleosome-excluding sequences, and γ hotspots are regions with high G + C content [2]. In *S. cerevisiae,* genome-wide mapping of meiotic hotspots revealed a strong correlation between DSBs and peaks of high G + C base composition [3]. To examine whether a similar correlation is also present in *C. neoformans,* the G + C content of the *MAT*α locus along with the 45-kb upstream and 40-kb downstream flanking regions was plotted with a 4-kb sliding window and 100-bp stepping for both serotype A and serotype D strains (Figure 7A; unpublished data). Different sizes of sliding window (1 kb, 5 kb, and 10 kb) were also used to conduct the analysis and the results from a 4-kb and a 5-kb sliding window looked very similar. Two major G + C peaks were identified in the *MAT* flanking regions in close proximity to the estimated hotspot windows (between 29 kb to 40 kb from the left border and 1 kb to 5 kb from the right border in H99), suggesting that these may represent *MAT*-associated γ hotspots.

To analyze further the correlation between G + C peaks and hotspot windows, we set the threshold of a G + C peak at 54% (6% higher than the average in this region), and calculated the overlap between the G + C peaks along with 2.5-kb flanking sequence and the potential hotspot regions to be 36.1%. The overlap percentage between two randomly drawn non-overlapping intervals was calculated to determine the statistical significance. This procedure was repeated 10,000 times and demonstrated that the correlation between the G + C peaks and hotspot windows observed is significant and not attributable to chance with a *p*-value of 0.049. This correlation between the G + C peaks and hotspot windows is also in accord with the finding that *MAT* heterozygosity is not required for hotspot activity.

A minor G + C peak in the *MAT* locus was observed in the promoter region between two divergently transcribed genes, *RPO41* and *BSP2,* and is present in three serotypes and both mating types (Figures 7A and S1). We propose that this may represent a gene conversion hotspot that could in part explain why these two genes were found to be ~99% identical between the two mating-type alleles in previous phylogenetic analyses [34].

### The G + C Rich Region Is Required for the Hotspot Activity

**Figure 8:**
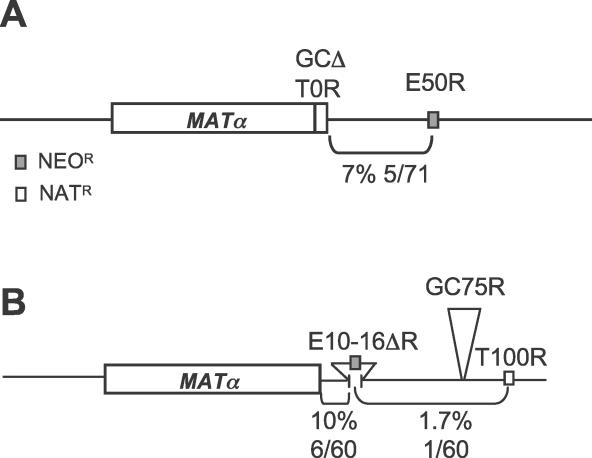
The G + C Rich Region Is Required but Not Sufficient for the Hotspot Activity Strains carrying G + C rich sequence deletion or insertion were generated and crossed to the wild-type strain KN99**a**. Recombination frequencies were scored and are depicted as percentages. (A) Deletion of the partial G + C rich region reduces recombination frequency to ~7%, near the genome-wide average. (B) Insertion of the partial G + C rich sequence at 75 kb from *MAT* does not increase recombination frequency between markers that are 10 kb and 100 kb from *MAT*.

Having established a correlation between the potential hotspots and a high G + C content, we next investigated whether the G + C rich sequence is required for activating recombination. The G + C peak on the right side of *MAT* encompasses a ~3.8-kb region, which includes the predicted gene *HOT1* and the first ~100 amino acids of the essential gene *NOG2.* To test the function of this region, we engineered a strain in the E50R background, in which 60% of the 5′ G + C peak sequence (2,282 bp) was deleted and replaced with a NAT^R^ marker (T0R), while the essential gene *NOG2* and its promoter region was left intact (Figure 7B). This strain was crossed to a wild-type *MAT* **a** strain and scored for meiotic recombination frequency. Among the 71 progeny analyzed, five of them were recombinant, indicating the recombination frequency has been reduced to ~7%, compared to the previous mapping result, in which a recombination frequency of ~17.5% was detected between markers T1R and E50R (Figures 4 and 8A). If deletion of the G + C rich region led to a complete loss of hotspot activity, recombination would be expected to occur at a frequency of ~3.7%. Thus, deleting 60% of the G + C rich sequence greatly reduced the recombination frequency to near the genome-wide average, but might not completely abolish hotspot activity, and in this context, the residual G + C rich sequences could still function to a limited extent.

To examine whether the deleted G + C rich region is sufficient to increase recombination when moved to another region of the genome, we amplified and targeted this 2.3-kb G + C rich region to a new location, ~75 kb away from the right border of *MAT* between the two dominant markers E10–16ΔR and T100R used in previous recombination experiments. A ~2.5% recombination frequency was observed between the two markers in the original strain background (Figure 3). If the introduced G + C rich region were sufficient to induce recombination, we would expect to observe increased recombination between the two markers. However, a recombination frequency of ~1.7% was detected; indicating that the activity of the *MAT*-linked hotspot is context-dependent and higher-order chromosomal structure may be involved in the regulation of the hotspot activity (Figure 8B). Alternatively, by being located within the vicinity of another functional hotspot, this may influence activity, or the G + C rich region that was moved lacks flanking sequences that are also required for activity.

## Discussion

In this paper we describe the discovery of *MAT*-associated meiotic recombination hotspots and potential REs in *C. neoformans.* We are certain that the recombination events detected are meiotic because of its life cycle—after the fusion of the two haploid parental nuclei in the basidium, meiosis immediately ensues and there are no mitotic divisions in between. In the *MAT* flanking region, recombination occurs almost 50 times more frequently than the genome-wide average (0.27 kb/cM versus 13.2 kb/cM or 3.7 cM/kb versus 0.076 cM/kb). We discuss how these hotspots may have impacted the evolution of the unusual *MAT* locus of C. neoformans and the implications of these findings with respect to mating-type switching in other fungal species.

### The Nature of the Hotspots

A correlation between the estimated hotspot locations and peaks of high G + C composition suggests these may represent examples of γ hotspots. Deletion of 60% of the G + C rich region reduces recombination frequency showing that it is required for hotspot activity. However, moving the G + C rich element to another location of the genome is not sufficient to induce recombination, which indicates that the *MAT*-linked hotspot activity may be context-dependent. This result is not surprising because studies on hotspot activity in both S. cerevisiae and S. pombe have drawn similar conclusions [42,43]. In both reports, the activity of hotspots was context-dependent and might involve higher-order chromosome structure or chromosome dynamics. Different levels of hotspot activity were observed when the hotspots were inserted at different genomic locations; furthermore, it was shown that the level of recombination activity is likely affected by the GC content of DNA sequences flanking the insertion [44].

In *S. cerevisiae,* of 177 hotspots identified throughout the genome 99 are associated with high G + C peaks, and this implies DSB distribution is affected by chromosome sequence and structure [3]. A similar association has also been described in humans [45]. The molecular mechanisms by which regions with rich G + C content lead to elevated recombination is proposed to involve accessible chromatin structures. DNA replication forks often transiently stall in G + C rich regions, which then require modification of histones to allow DNA replication to proceed. This local modification of histones may render the DNA more accessible to the recombination machinery to initiate DSB formation [5]. In addition to the two G + C peaks located outside of *MAT,* we also identified a moderate G + C peak within the *MAT* locus itself (Figure 7A). This peak is located in the intergenic region between two divergently transcribed genes, *RPO41* and *BSP2* and is present in all *MAT* **a** and *MAT*α alleles that have been sequenced in three different serotypes of *Cryptococcus* strains (Figure S1). Below, we discuss how this region, along with the *MAT*-associated hotspots, might have affected the evolution of the *MAT* locus in *Cryptococcus.*

How might these recombinational activators function at a mechanistic level? Given the G + C rich nature of the regions of the genome in which they lie, our working model, mostly based on studies from *S. cerevisiae,* is that the chromatin structure is locally altered, facilitating access and cleavage by an endonuclease and other associated proteins that are required for recombination. At present, the inability to conduct synchronous meiosis in this organism will make it experimentally challenging to define the inciting recombinogenic lesions (such as nicks or DSBs) at a molecular level. Further advances will require an understanding of the signals that control the formation of the basidium fruiting structure, in which nuclear fusion and meiosis occur in the traditional **a**-α sexual cycle, and of the signals that stimulate meiosis.

### Exchanging *MAT* onto Another Genetic Background

Our segregation analysis showed that the occurrence of DCOs on both sides of *MAT* is ~4 times higher than expected (Figure 5). This negative interference effect, although uncommon, has been described in relation to the *am* locus in Neurospora crassa and at a translocation breakpoint in maize [46,47]. In our study, the negative interference effect between the *MAT*-associated hotspots increases the frequency of exchanging the *MAT* locus onto another genetic background. What causes this negative interference effect? One interpretation is that when a crossover occurs on one side of *MAT* this may help align the heterozygous *MAT* region, and thereby, facilitate a second crossover on the other side of *MAT*. Alternatively, it could be a result of the presence of a subset of population which has globally “hotter” recombination than the reminder of cells in the population [48].

In a population where the **a** and α cells have divergent backgrounds, nucleotide divergence might limit the efficiency of meiotic recombination as mismatch repair acts to limit homologous recombination. REs that stimulate exchange of *MAT* could help maintain sexual potential by promoting its transposition onto a divergent genetic background. Alternatively, in highly related inbreeding populations, this mechanism may work to ensure isolates of both mating types are maintained. For example, our previous studies on the Cryptococcus gattii population showed that several of the VGI isolates are identical based on multilocus sequence typing analysis, except for the *MAT* locus [49]. This could be an example in which the recombination activators have served to exchange only *MAT* to result in a highly inbred background to retain the ability to produce spores via **a** × α mating.

### Hotspot Activity and the Evolution of *MAT*

In higher eukaryotes, sexual identity is controlled by sex chromosomes, which are large, complex structures that account for a significant portion of the genome. In fungi, such as *S. cerevisiae,* the sex-determining region represented by *MAT* has a simpler structure and is less than 1 kb in length. Previous studies on the structure of the *MAT* locus in C. neoformans have provided insight as to how this unusual fungal genomic sex-determining region evolved, and the hypothesized events resemble the evolutionary steps thought to have shaped the sex chromosomes in animals and plants [50]. Evidence suggests that the *C. neoformans MAT* locus evolved from an ancient tetrapolar mating system through four major steps. First, a series of genes were acquired into the two ancestral *MAT* loci, forming two unlinked gene clusters. Second, these two loci then were fused via chromosomal translocation and resulted in a tripolar intermediate state. Third, the tripolar system collapsed to a bipolar one via interallelic recombination, and fourth, ongoing gene conversions and inversions rearranged the *MAT* loci into the current alleles [10,34].

What roles do hotspots serve in terms of the evolution of *MAT*? We propose that one important function is to prevent the *MAT* locus from expanding and eventually capturing the entire chromosome. By increasing recombination in the *MAT* flanking regions, the accumulation of sequence differences is restricted between the **a** and α alleles. Furthermore, if any transposon locally transposed into the *MAT*-flanking region from within *MAT,* recombination hotspots would essentially sweep the transposon away to protect the boundaries of *MAT* by enabling recombination to occur between *MAT* and the transposed element in meiotic progeny.

**Figure 9:**
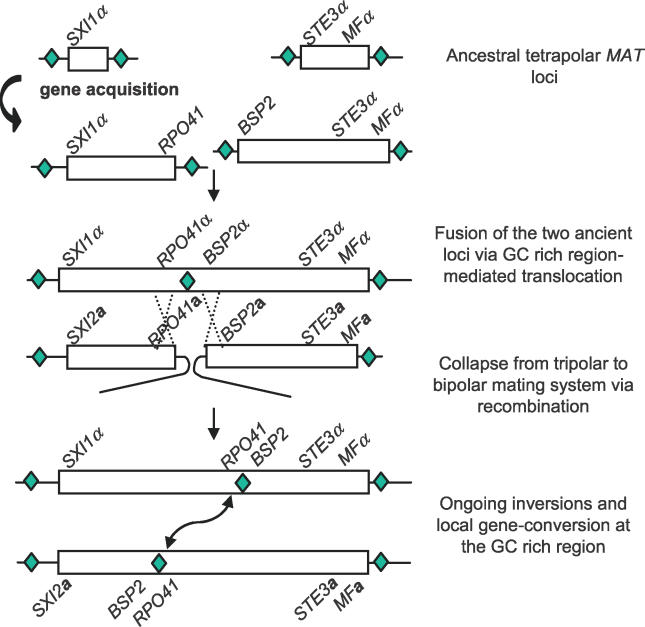
A Model of How the *MAT*-Associated Hotspots Influenced the Evolution of *MAT* in C. neoformans The ancestral tetrapolar *MAT* loci encode homeodomain protein genes (e.g., *SXI1*α) and the pheromones and pheromone receptors (e.g., *STE3*α and *MF*α). Diamonds flanking *MAT* represent G + C rich recombination hotspots. Hypothesized evolutionary steps include gene acquisition, translocation, locus collapse, and ongoing gene conversion and inversions.

Recombination hotspots likely contributed to several of the hypothesized steps [34] in the *Cryptococcus MAT* locus evolutionary model (Figure 9). First, in the earliest steps when sequential gene acquisition occurred in the ancestral *a* and *b* loci of a tetrapolar system, hotspots would have facilitated capture of genes into both mating-type alleles by enabling rapid assimilation of the flanking region into both alleles before the linked genes were captured by inversions into the *MAT* locus. This also could be the case in *C. albicans,* in which several additional genes *(PIK1, OBP1,* and *PAP1)* have been recruited into both the **a** and α alleles of the *MTL* locus [51]. Second, we hypothesize that the G + C rich region in the intergenic region between the *RPO41* and *BSP2* genes could also be a hotspot that impacted the fusion of the ancestral tetrapolar loci to generate the tripolar intermediate. We hypothesize that genes that fall into the recent gene category in the previous phylogenetic analysis [34] flanked the ancestral tetrapolar *MAT* loci, which were surrounded by four G + C rich domains on both boundaries (Figure 9). The G + C rich activators might have first served to facilitate the fusion of the two tetrapolar *MAT* loci. After formation of a single contiguous *MAT* locus, the G + C rich region in the intergenic region of the *RPO41* and *BSP2* genes would further serve as a gene conversion hotspot, contributing to render these genes serotype- rather than mating-type-specific, as observed, via more recent and ongoing local gene conversion events [34].

Examining the junctions of the *MAT* locus from different lineages showed possible traces for the consequences of hotspot activation that also contributed to remodel *MAT,* possibly via aberrant events. For example, the *IKS1, BSP3,* and *NCM1* genes are integral components of *MAT* in var. *grubii* and *gattii,* but these linked genes have been rendered homozygous and have become three adjacent common flanking genes in var. *neoformans* and therefore represent examples of gene eviction events. Furthermore, phylogenic analysis revealed that the *IKS1, BSP3,* and *NCM1* alleles in var. *neoformans* descend the ancestral α alleles [34]. One can imagine that this could have resulted from a gene conversion triggered by a DSB that occurred near the hotspot at the telomere-distal end of *MAT* (Figure S2). Although our results suggest that the *MAT*-associated recombination events we detect are more likely to be crossover events, studies in S. cerevisiae demonstrate that crossover hotspots are also gene conversion hotspots [52]. Sequence analysis suggests that a deletion may have also occurred at the telomere-proximal border of *MAT* in a serotype C isolate, and this may represent an aberrant event involving repair of an inciting lesion emanating from the recombinational activator flanking the telomere-proximal border of *MAT* (J. Fraser and J. Heitman, unpublished data).

Another example of the potential evolutionary impact of the *MAT*-associated hotspots is an interspecies DNA transfer event between the two varieties. A recent report showed that an ~40-kb genomic region that is in the subtelomeric region on the *MAT* chromosome of var. *grubii* was transferred to a non-homologous chromosome in var. *neoformans* strains [53]. This region of the genome, the identity island, shares 98.5% nucleotide identity and is the most closely related region of the genome between the otherwise divergent var. *grubii* and var. *neoformans* lineages, which share an average of ~85%–90% nucleotide identity. Thus, this was the last region of DNA exchange between the two as they diverged, and this event involved an intermediate AD hybrid produced by intervarietal mating. We hypothesize that a DSB generated in the telomere-proximal *MAT*-associated hotspot might have facilitated this inter-species genome transfer, in addition to the presence of Cnl1 repetitive elements located at the sites of the chromosomal translocation.

### Relevance to *MAT*-Associated Recombination in Model and Pathogenic Yeast

It is relevant to consider our findings with respect to *MAT*-linked recombination in C. neoformans in light of previous and recent studies on *MAT* switching or recombination in other yeasts. Studies have shown that gene conversion at the *MAT* locus naturally occurs at a low frequency in S. cerevisiae **a**/α diploid strains. For example, in a standard mating assay one can readily detect rare *MAT* homozygosis events that occur in which **a**/α strains become **a**/**a** or α/α by mitotic gene conversion. In now classic studies, a marker tightly linked to *MAT, cry1,* which confers recessive resistance to cryptopleurine, was employed to identify *MAT* homozygous diploids to perform complementation tests of sterile mutations that could not be scored in a diploid [54]. In contrast to mating-type switching, these events involve only the active *MAT* locus, without participation of the Ho endonuclease or the silent mating-type cassettes.

A second example of recombination at the mating-type locus occurs in *Candida albicans,* a diploid pathogenic yeast. The fungus maintains the *MTL* locus in an **a**/α configuration with no silent mating-type cassettes, and homozygosis of *MTL* is required to produce **a/a** and α/α mating-competent isolates and enable the white to opaque cell-type switch necessary for efficient mating [55,56]. While growth on sorbose selects for complete loss of Chromosome 5 harboring the *MTL* locus [57] in naturally occurring** a**/**a** and α/α isolates, it has recently been discovered that homozygosis of the *MTL* locus has occurred via a local gene conversion event that enables the cell to become mating-competent (D. Soll, personal communication; Figure 9). This implies that a DSB or other lesion might be created close to the border of *MTL* to initiate gene conversion. Given that C. albicans contains only an active *MTL* locus with **a** or α alleles, and no silent cassettes, this gene conversion evoked mating-type switch more closely parallels our observation in *C. neoformans MAT*-linked recombination compared to the endonuclease or DNA lesion provoked gene conversion from silent to active *MAT* cassettes in S. cerevisiae and S. pombe (Figure 9).

### *MAT*-Associated Recombination and Evolution of Homothallism and Heterothallism

The presence of recombinational activators linked to mating-type loci could also have influenced the common evolutionary transitions that occur from homothallism to heterothallism and from heterothallism to homothallism in the fungal kingdom, and between tetrapolar and bipolar mating-type systems. For example, in the model fungi *Ustilago maydis, Coprinus cinereus,* and *Schizophyllum commune,* which all have heterothallic tetrapolar mating systems, one locus encodes pheromones and pheromone receptors and the other encodes two divergently transcribed homeodomain proteins, and the products of these alleles are not intercompatible with each other and only serve to activate the products of an opposite allele of the mating-type locus [58]. However, in a homothallic ancestor, compatible homeodomain proteins may have been expressed from a common locus, or self-compatible pheromones and pheromone receptors from the other mating-type locus. The presence of recombinational activators could have served to recombine two self-compatible loci to produce two novel recombinant loci that were then no longer self-compatible, but only compatible with each other, affecting a switch from homothallism to heterothallism. Transitions in the opposite direction, from heterothallism to homothallism could also readily occur via such a mechanism. Finally, in the two examples in which a tetrapolar system has collapsed to a bipolar system that are understood in molecular detail, C. neoformans and Ustilago hordei [59], recombinational activators could have subserved a role in triggering the chromosomal translocation events that linked the two previously distant mating-type alleles into one contiguous region. Further studies will be required to address whether recombinational activators might flank or lie within the *a* and the *b* loci of the tetrapolar mating-type systems in *U. maydis, C. cinereus,* and *S. commune,* although the current literature does not imply the existence of recombinational activators in the *A* locus of C. cinereus [60]. However, a high frequency of recombination is observed in the *B* locus of C. cinereus and S. commune and in the *a* locus of *U. maydis,* which suggests that a higher rate of recombination outside of a *MAT* locus could be a general feature in the basidiomycetes [61]. Finally, in the ascomycete phylum of fungi, well documented examples of transitions from heterothallism to homothallism have involved either fusion or linkage of opposite mating-type alleles *(Cochliobolus),* or the simultaneous presence of both mating-type alleles at unlinked regions of the genome *(A. nidulans)* [62,63]. While it is not known if recombinational activators are linked to the mating-type locus in these fungi, if such a mechanism were to operate, it might have contributed to these dramatic transitions in the function and organization of the mating-type determinants in these systems.

### The Implication of *MAT*-Associated Recombination Hotspots on the Origins of Metabolic and Biosynthetic Gene Clusters

While clustering into operons of genes encoding functionally related proteins is a common feature of prokaryotes, increasing evidence now supports the idea that gene order is also a non-random feature of eukaryotic genomes, and genes encoding related functions are often physically clustered [64]. In fungi, several gene clusters involved in the production of secondary metabolites and biosynthetic pathways have been identified and characterized [65–67]. How these clusters are assembled during evolution is unclear. It has been shown that some gene clusters are meiotic recombination coldspots, which is also a characteristic of the *MAT* locus. Coincidentally, we found that the regions flanking the *DAL* gene cluster for allantoin utilization in S. cerevisiae are increased for meiotic recombination based on previous studies [66]. Our hypothesis on the evolution of *C. neoformans MAT* gene cluster formation may provide clues to understand how gene clusters evolve in general, as common mechanisms may operate during evolution that are fashioned different gene clusters.

**Figure 10:**
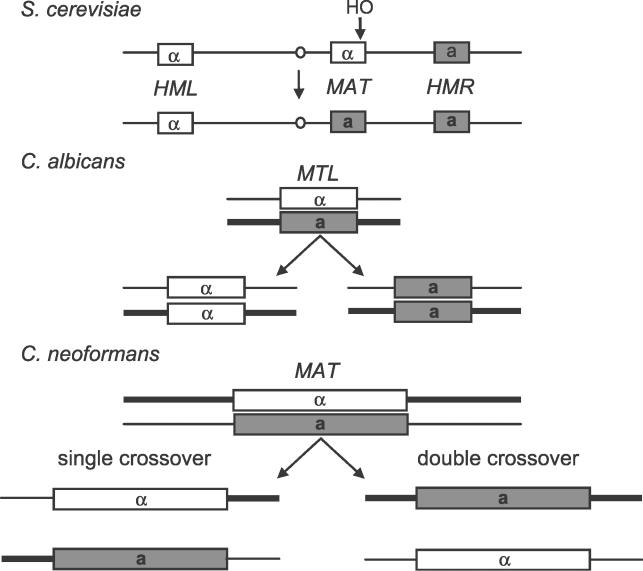
Mating-Type Switching and Recombination Associated with Mating-Type Loci in Model and Pathogenic Yeasts In *S. cerevisiae,* mating-type switching is triggered by a DSB generated at the *MAT* locus by the HO endonuclease, and a gene conversion event occurs between *MAT* and a silent cassette, usually the one which encodes the opposite mating type. In the diploid human fungal pathogen *C. albicans,* local gene conversion at the *MTL* locus leads to the formation of *MTL* homozygous isolates that can be mating-competent. In *C. neoformans,* the *MAT*-associated recombination hotspots induce SCO or DCO events in the *MAT* flanking regions that facilitate exchange of *MAT* onto different backgrounds among the meiotic progeny.

In summary, we have identified *MAT*-associated recombination hotspots in C. neoformans and established a link between the location of these hotspots and G + C rich regions of the genome. These hotspots are hypothesized to have impacted the evolution of *MAT* in several aspects. It will be of interest to ascertain if similar elements operate in the *MAT* loci of other basidiomycetes as a general phenomenon by which *MAT*-associated recombination hotspots drive evolution or inheritance of the *MAT* locus. Finally, as illustrated in Figure 10, recombination around the *MAT* loci in the ascomycetous fungi and in *C. neoformans* is achieved via different mechanisms, providing conclusive evidence for independent evolutionary events that nevertheless affect these loci in similar ways. In particular, in our finding of *MAT*-associated recombination in C. neoformans and the origin of mating-competent *MTL* homozygous strains of *C. albicans* via similar concerted gene conversion events, we observe a striking parallel in two divergent, common, successful human fungal pathogens.

## Materials and Methods

### Strains and media.

All C. neoformans strains used in this study (listed in Table S1) were generated in the reference congenic strains JEC21 (α)/JEC20 (**a**) (serotype D) and H99 (α)/KN99 (**a**) (serotype A) backgrounds [36,68,69]. Strains were grown on yeast extract-peptone-dextrose (YPD) and synthetic (SD) media, and mating assays were conducted on 5% V8 juice agar medium in the dark at room temperature on plates without parafilm to limit CO_2_ accumulation and with the agar side down and cells facing up to promote desiccation [36,70].

### Insertion of dominant selectable markers, and the deletion and insertion of a G + C rich region into the *MAT* chromosome.

NAT^R^ (Nourseothricin acetyl-transferase) or NEO^R^ (Neomycin phosphotransferase II) dominant selectable markers were integrated into various locations on the *MAT* chromosome through homologous recombination of overlap PCR-generated constructs as described previously [71–73]. Briefly, the 5′ and 3′ regions of the insertion site were PCR-amplified with region-specific primers (Table S2) and the NAT^R^ or NEO^R^ dominant selectable markers were amplified with the universal M13F and M13R primers. Next, overlap PCR was performed with the dominant marker, and the 5′ and 3′ flanking DNA as templates and amplified with region-specific primers. The overlap PCR products were purified and introduced into the desired strains by biolistic transformation [73,74]. Stable transformants were selected on YPD medium containing nourseothricin (100 mg/L) or G418 (200 mg/L). Genomic DNA was extracted and initially screened by diagnostic PCR, and positive clones were further characterized by Southern blot analysis to confirm the desired insertion.

A strain (YPH296) comprising a deletion of the G + C rich region was created in the YPH3 background using the above described method, except that Takara *LA Taq,* which is designed for high G + C content PCR, was used in the PCR reactions to amplify the DNA. To insert the 2.3-kb G + C rich region 75 kb away from the right *MAT* locus border, strain YPH29 was selected on 5-FOA plates to obtain the *ura* ^−^ strain YPH297. Next, a four-piece overlap PCR construct including the 5′ flanking DNA, *URA5* marker, the 2.3-kb G + C rich region and the 3′ flanking DNA were amplified and transformed into strain YPH297 to introduce the 2.3-kb G + C rich sequence at the location 75 Kb away from the *MAT* border (strain YPH306).

### Micromanipulation of meiotic basidiospores and segregation analysis.

Mating reactions of the desired strains were established by co-culturing the opposite mating-type cells on V8 media (pH 5 or pH 7 for serotype A or serotype D strains, respectively). Matings were conducted at room temperature in the dark for 2–4 wk until robust filamentation and sporulation were observed by microscopy. The filaments and the basidiospores on the edges of a mating patch were removed on an agar plug taken with a glass Pasteur pipet and transferred to a YPD plate. The plate was then placed on a micromanipulator and the basidiospores were randomly selected with a 25-μm microneedle (Cora Styles Needles ‘N Blocks, Dissection Needle Kit) and arranged in a grid on the surface of the medium. For a more detailed description of micromanipulation, see [70]. Each basidium fruiting structure harbors a single diploid nucleus, which undergoes a single round of meiosis, and then repeated rounds of mitosis give rise to chains of >10 basidiospores. As it is extremely technically challenging to dissect from individual basidia, random spore analysis was conducted. Isolated basidiospores were incubated at 30 °C for 2–3 d to allow the spores to germinate, and the resulting yeast colonies were subcultured to fresh YPD medium. The mating-type and dominant selectable markers of each segregant were determined by crossing the segregants to reference strains JEC20 (**a**) and JEC21 (α) and by growth assays on YPD medium supplemented with nourseothricin or G418.

### Construction of **a**/**a** and α/α homozygous and **a**/α heterozygous diploid strains.

Strains YPH38 (*MAT*α NEO50R) and YPH52 (*MAT*α NAT50L), which have integrated dominant selectable markers, were generated in the congenic JEC21 and JEC20 serotype D strain background by the methods described above. To introduce an identical *ura5* allele into these strain backgrounds, strains YPH38 and YPH52 were crossed to strain YPH50 *(MAT* **a** *ura5)* and progeny containing the dominant selectable marker and the *ura5* allele were isolated.

The** a**/**a** diploid strain YPH59 was obtained by fusing strains YPH54 *(MAT* **a** NEO50R *ura5)* and YPH57 *(MAT* **a** NAT50L *ura5);* the α/α diploid strain YPH58 was isolated by the fusion of strains YPH55 *(MAT*α NEO50R *ura5)* and YPH56 *(MAT*α NAT50L *ura5);* the **a**/α diploid strain YPH62 was isolated by the fusion of strains YPH55 *(MAT*α NEO50R *ura5)* and YPH57 *(MAT* **a** NAT50L *ura5).* Fusion between cells of the same mating type was assisted with an opposite mating-type pheromone donor strain which does not harbor any dominant selectable marker (ménage a trois matings). All diploid strains were first identified according to their NAT^R^ and NEO^R^ double resistance and then confirmed by FACS analysis to confirm a 2n/4n content of DNA as described [75]. To facilitate the generation of meiotic progeny via monokaryotic fruiting from these *MAT*-homozygous diploids, the *SXI1α* and *SXI2**a*** expression vectors pCH258 and pCH285 [28] were transformed by electroporation into strains YPH59 (**a**/**a**) and YPH58 (α/α) respectively, and Ura^+^ transformants were selected to generate strains YPH69 (**a**/**a +** *SXI1α*) and YPH63 (α/α + *SXI2* ***a***). The diploid strains were incubated on V8 medium and the segregants were analyzed by the methods described above.

### Identification of polymorphisms between the parental strains used for the generation of congenic strain pair H99 and KN99a.

Polymorphisms between the parental strains used for the generation of congenic strain pair H99 and KN99**a** were identified via three different approaches. First, the 10-kb upstream and downstream flanking sequences of the *MAT* **a** allele from strain 125.91 were retrieved from Genbank and compared to the H99 genome to identify polymorphisms. Two other approaches were used to identify polymorphisms that lie beyond a 10-kb upstream and downstream sequence of the *MAT* locus. First, shot-gun sequencing of a genomic DNA library of strain KNA14 [36] was performed and the retrieved sequence data were compared to H99 (K. Nielsen, J. Fraser, and F. Dietrich, personal communication). Second, PCR and sequencing of selected *MAT* flanking regions in strain KNA14 were also conducted to identify polymorphisms.

### Oligonucleotide primers and PCR experiments.

Primers used to identify polymorphisms in the backcross strains are listed in Table S2, and the region-specific primers used for strain construction are listed in Table S3. Takara *Ex Taq* was used in PCR following the conditions recommended by the manufacturer.

## Supporting Information

Figure S1 A Minor G + C Peak Is Detected between *RPO41* and *BSP2* in All *MAT* Loci Sequenced JEC21 is a serotype D *MAT*α strain; WM276 is a serotype B *MAT*α strain; 125.91 is a serotype A *MAT* **a** strain; E566 is a serotype B *MAT* **a** strain. The left arrows indicate the position of the minor G + C peaks, and the arrows on the right present the major G + C peaks on the right side of *MAT*. In the serotype D lineage, three genes *(IKS1, BSP3,* and *NCM1)* have been evicted from *MAT* and have become common flanking genes in JEC20 and JEC21. Therefore the major G + C peak in these two strains is more distant from the *MAT* boundary when compared to the other two lineages (see Discussion).
(50 KB PDF)

Figure S2 A Model of How the *MAT*-Associated Hotspots Contribute to Gene Eviction Events to Remodel *MAT* in C. neoformans
A DSB that occurred near the hotspot at the telomere-distal end of *MAT* triggered gene conversion of genes *IKS1, BSP3, NCM1,* and *ETF1;* gene inversion subsequently occurred at *ETF1* allele in the *MAT*α locus. These events made *IKS1, BSP3,* and *NCM1* become three adjacent common flanking genes in var. *neoformans*.
(43 KB PDF)

Table S1 Strains Used in This Study (92 KB DOC)

Table S2 Primers Used to Identify Polymorphisms in the Backcross Strains (48 KB DOC)

Table S3 Primers Used for Strain Construction (78 KB DOC)

## Abbreviations

bp: base pair
DCO: double crossover
DSB: double-stranded DNA break
kb: kilobase
*MAT*: Mating-type locus
RE: recombinational enhancer
SCO: single crossover
