# Supplementary material for: Recombination Hotspots Flank the Cryptococcus Mating-Type Locus: Implications for the Evolution of a Fungal Sex Chromosome
Source: PLoS Genet. 2006 Nov 3;2(11):e184. doi: 10.1371/journal.pgen.0020184 (PMC1630710; doi:10.1371/journal.pgen.0020184)
Supplement: Supplementary file 1 [file pgen.0020184.sg001.pdf]

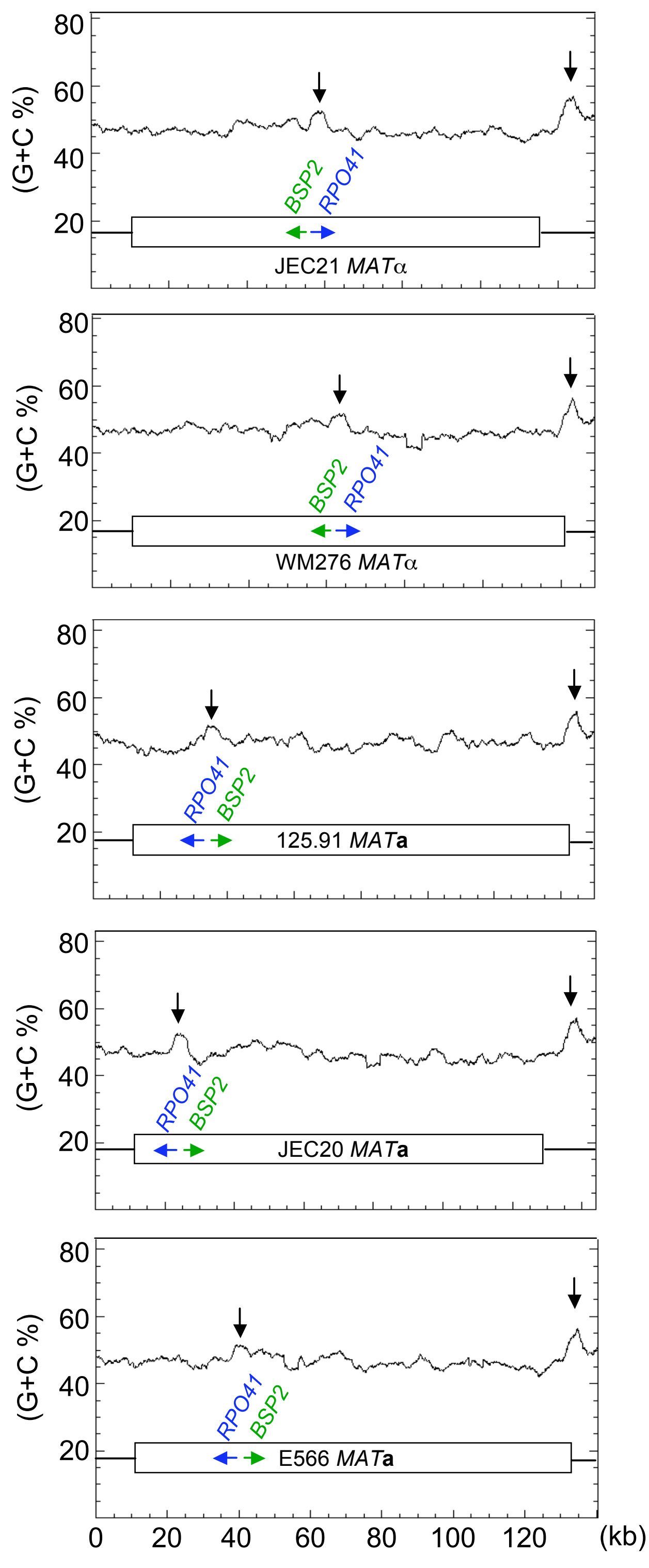

80
60
40
20
(G+C %)
BSP2
RPO41
JEC21 MATα
WM276 MATα
125.91 MATa
JEC20 MATa
E566 MATa
0
20
40
60
80
100
120
(kb)
