# Supplementary material for: Recombination Hotspots Flank the Cryptococcus Mating-Type Locus: Implications for the Evolution of a Fungal Sex Chromosome
Source: PLoS Genet. 2006 Nov 3;2(11):e184. doi: 10.1371/journal.pgen.0020184 (PMC1630710; doi:10.1371/journal.pgen.0020184)
Supplement: Supplementary file 2 [file pgen.0020184.sg002.pdf]

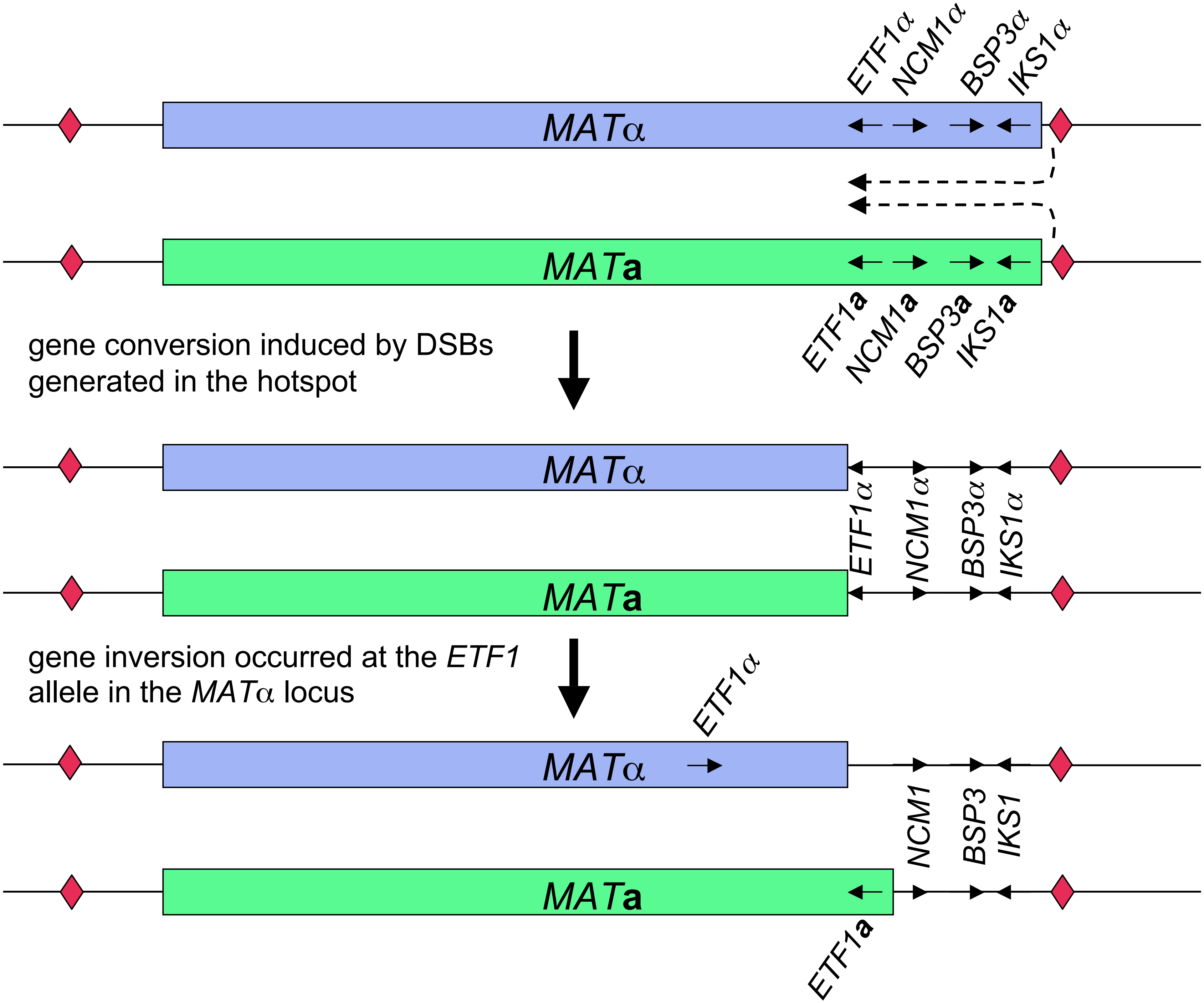

ETF1α
NCM1α
BSP3α
IKS1α
MATα
MATa
ETF1a
NCM1a
BSP3a
IKS1a
gene conversion induced by DSBs
generated in the hotspot
MATα
ETF1α
NCM1α
BSP3α
IKS1α
MATa
gene inversion occurred at the ETF1
allele in the MATα locus
ETF1α
MATα
NCM1
BSP3
IKS1
MATa
ETF1a
