# Supplementary material for: Recombination Hotspots Flank the Cryptococcus Mating-Type Locus: Implications for the Evolution of a Fungal Sex Chromosome
Source: PLoS Genet. 2006 Nov 3;2(11):e184. doi: 10.1371/journal.pgen.0020184 (PMC1630710; doi:10.1371/journal.pgen.0020184)
Supplement: Supplementary file 3 [file pgen.0020184.st001.doc]

# Supporting information

Table S1. Strains used in this study

| Strain | Genotype | Serotype | Source |
| --- | --- | --- | --- |
| H99 | *MAT*α | A | [76] |
| KN99**a** | *MAT***a** | A | [36] |
| AI24 | *MAT*α T77L | A | This study |
| AI32 | *MAT***a** E44L T77L | A | This study |
| YPH3 | *MAT*α E50R | A | This study |
| YPH5 | *MAT*α T100R | A | This study |
| YPH17 | *MAT***a** E50R | A | This study |
| YPH28 | *MAT*α T10-16ΔR E50R | A | This study |
| YPH29 | *MAT*α E10-16ΔR T100R | A | This study |
| YPH31 | *MAT*α T1R E50R | A | This study |
| YPH32 | *MAT*α T5R E50R | A | This study |
| YPH33 | *MAT*α E1R T100R | A | This study |
| YPH34 | *MAT*α E5R T100R | A | This study |
| YPH296 | *MAT*α GCΔ T0R E50R | A | This study |
| YPH297 | *MAT*α E10-16ΔR T100R *ura5* | A | This study |
| YPH306 | *MAT*α E10-16ΔR GC 75R T100R | A | This study |
| JEC21 | *MAT*α | D | [77] |
| JEC20 | *MAT***a** | D | [77] |
| YPH38 | *MAT*α E50R | D | This study |
| YPH50 | *MAT***a** *ura5* | D | This study |
| YPH51 | *MAT*α *ura5* | D | This study |
| YPH52 | *MAT***a** T50L | D | This study |
| YPH54 | *MAT***a** E50R *ura5* | D | This study |
| YPH55 | *MAT*α E50R *ura5* | D | This study |
| YPH56 | *MAT*α T50L *ura5* | D | This study |
| YPH57 | *MAT***a** T50L *ura5* | D | This study |
| YPH58 | *MAT*α E50R *ura5* /*MAT*α T50L *ura5* (diploid) | D | This study |
| YPH59 | *MAT***a** E50R *ura5* /*MAT***a** T50L *ura5* (diploid) | D | This study |
| YPH62 | *MAT*α E50R *ura5* /*MAT***a** T50L *ura5* (diploid) | D | This study |
| YPH63 | *MAT*α E50R *ura5* /*MAT*α T50L *ura5* + *SXI2****a*** | D | This study |
| YPH69 | *MAT***a** E50R *ura5* /*MAT***a** T50L *ura5* + *SXI1α* | D | This study |
