# Supplementary material for: Recombination Hotspots Flank the Cryptococcus Mating-Type Locus: Implications for the Evolution of a Fungal Sex Chromosome
Source: PLoS Genet. 2006 Nov 3;2(11):e184. doi: 10.1371/journal.pgen.0020184 (PMC1630710; doi:10.1371/journal.pgen.0020184)
Supplement: Supplementary file 4 [file pgen.0020184.st002.doc]

Table S2. Primers used to identify polymorphisms in the backcross strains

| Primer | Sequence (5' to 3') | Position of the polymorphisms |
| --- | --- | --- |
| JOHE10928 | GTTTATGATCCATCCGCAGG | R 2 kb |
| JOHE10929 | CCACGAGAAGCCGGTCATCT | R 2 kb |
| JOHE10940 | AACGAGGACGATCTAGTTGG | R 10 kb |
| JOHE10941 | AAGTTCCGGCACGAGCGTTG | R 10 kb |
| JOHE11171 | CCAAGACTTGATGGCTTGAG | R 20 kb |
| JOHE11172 | CACTACTCCTATGGACCCTC | R 20 kb |
| JOHE11173 | CAGAGGCTTCAGTGGCATTC | R 30 kb |
| JOHE11174 | CTGCTGCGAGGACTTATATG | R 30 kb |
| JOHE11490 | GCTCGATCACCATCAAACCC | L 2 kb |
| JOHE11491 | TCTGGCATCCATGGCCGC | L 2 kb |
| JOHE11492 | GCGCCATTGTCATGTTACCG | L 40 kb |
| JOHE11493 | GGAAGGAGAGTGGTGTCTCG | L 40 kb |
| JOHE11494 | CAAGGAACCGGCAAGCTTTC | L 60 kb |
| JOHE11495 | TCACTGAGGACTTTGATGCC | L 60 kb |
| JOHE11496 | GATGCTCCCTGTGCTGCGAG | R 16 kb |
| JOHE11497 | CAACACATCAGCCATCCAGC | R 16 kb |
| JOHE11498 | AGGAATATGCGGCAGCTGTTG | R 36 kb |
| JOHE11499 | GATTTGGTTTCACCGCTCTGG | R 36 kb |
| JOHE14032 | CTCGTTCGTAACTGGTAAGG | L 13 kb |
| JOHE14033 | CCCACTCAAAGTCCACAACA | L 13 kb |
| JOHE17439 | CCGATACGGACTCTGCCCCG | L 29 kb |
| JOHE17441 | TTCTGCATCTACATCTTCCCC | L 29 kb |
