# Supplementary material for: Recombination Hotspots Flank the Cryptococcus Mating-Type Locus: Implications for the Evolution of a Fungal Sex Chromosome
Source: PLoS Genet. 2006 Nov 3;2(11):e184. doi: 10.1371/journal.pgen.0020184 (PMC1630710; doi:10.1371/journal.pgen.0020184)
Supplement: Supplementary file 5 [file pgen.0020184.st003.doc]

Table S3. Primers used for strain construction

| Primer | Sequence (5' to 3') | Marker |
| --- | --- | --- |
| JOHE8689 | CAAAGTTGGGCTTCGGTGGC | T77L |
| JOHE8719 | GCCAAGTCAGTATCTGTGGCTGCGAGGATGTGAGCTG | T77L |
| JOHE8720 | AGCTCACATCCTCGCAGCCACAGATACTGACTTGGC | T77L |
| JOHE8721 | TAGTTTCTACATCTCTTCCGGCAAGTCAATACATTC | T77L |
| JOHE8722 | GAATGTATTGACTTGCCGGAAGAGATGTAGAAACTAG | T77L |
| JOHE8690 | AGCAAGACCAACTGTAAGCGG | T77L |
| JOHE9284 | CTATCTCCCATACATTCCCG | E44L |
| JOHE9285 | CAAAATGAGCACTCCTCCGCTGCGAGGATGTGAGCTG | E44L |
| JOHE9286 | CAGCTCACATCCTCGCAGCGGAGGAGTGCTCATTTTG | E44L |
| JOHE9287 | CTAGTTTCTACATCTCTTCAGACTGGGAAAGAGAGGC | E44L |
| JOHE9288 | GCCTCTCTTTCCCAGTCTGAAGAGATGTAGAAACTAG | E44L |
| JOHE9289 | ATAAGAGTGTACCAACGGTC | E44L |
| JOHE10895 | acgtcttctttggtagatcc | T100R |
| JOHE11196 | CTGGCCGTCGTTTTACAgtgctggctcattgttg | T100R |
| JOHE11197 | GTCATAGCTGTTTCCTGggccctcgatgattgacg | T100R |
| JOHE10898 | tagtcatcgccaacagagtctgc | T100R |
| JOHE10899 | tgcgtccgatggatcgtc | E50R |
| JOHE11198 | CTGGCCGTCGTTTTACAgactcgttgactgttgtt | E50R |
| JOHE11199 | GTCATAGCTGTTTCCTGgatgtgaacaagaagttc | E50R |
| JOHE10902 | catctcctacaaggaggc | E50R |
| JOHE11858 | CTCGATCGAATACCTCGACTG | 10-16Δ R |
| JOHE11859 | CTGGCCGTCGTTTTACACCAGTGTGGCCAGCGGTATC | 10-16Δ R |
| JOHE11860 | GTCATAGCTGTTTCCTGCTGTAGATTGCGCTTTGATG | 10-16Δ R |
| JOHE11861 | ACGAACCTAGGTATGAGAGC | 10-16Δ R |
| JOHE13395 | CACGTATGTTTAGATCGGAG | E1R |
| JOHE13396 | CTGGCCGTCGTTTTACAGGTGCACCAGAGACATTCGA | E1R |
| JOHE13397 | GTCATAGCTGTTTCCTGCGTCCGCCCGACTACTTTCT | E1R |
| JOHE13398 | CGTCGCTGTGCCTCCGCTCC | E1R |
| JOHE13399 | GCTCCTCCGTCAGTTCTCTG | T5R |
| JOHE13400 | CTGGCCGTCGTTTTACACATCTTCGTCATCCTCGTCG | T5R |
| JOHE13401 | GTCATAGCTGTTTCCTGTACCCTCAGCGAAGCAACTT | T5R |
| JOHE13402 | CGTGCAGCGCACCCATCTAA | T5R |
| JOHE15227 | ctgtaagtcttcaaatcacc | T50L |
| JOHE15228 | CTGGCCGTCGTTTTACAGCCTTACCACTCGTCGTACG | T50L |
| JOHE15229 | GTCATAGCTGTTTCCTGATCGGAGTTCACGAAACTGT | T50L |
| JOHE15230 | CCGTTGAGGTCAAGGATTTG | T50L |
| JOHE17587 | AACAGCCCCAGCCCATTGTAAC | GCΔT0R |
| JOHE17588 | CTGGCCGTCGTTTTACATCAACTTCGCAACGAGCACC | GCΔT0R |
| JOHE17589 | GTCATAGCTGTTTCCTGGGCAATCAGCAAGAGGGATAGC | GCΔT0R |
| JOHE17590 | TGGGTGGGTGTCAGTATGGAAGTG | GCΔT0R |
| JOHE17552 | CGATGAACCCAAGCAGTTTTTC | GC75R |
| JOHE17553 | CTGGCCGTCGTTTTACAGAAGTTGACGAAGATGATGGTGC | GC75R |
| JOHE17643 | GTCATAGCTGTTTCCTGGGTGCTCGTTGCGAAGTTGAT | GC75R |
| JOHE17644 | CTGGCCGTCGTTTTACAGGTTGTCTATTCATGAGATGC | GC75R |
| JOHE17645 | TGTAAAACGACGGCCAGTGGTTCATCTACTGAGTCTCCCCAC | GC75R |
| JOHE17555 | GAGGATTATGGTTGTTGAGGCG | GC75R |
